# Salidroside alleviates oxidative stress in the liver with non- alcoholic steatohepatitis in rats

**DOI:** 10.1186/s40360-016-0059-8

**Published:** 2016-04-14

**Authors:** Ze-ran Yang, Hui-fang Wang, Tie-cheng Zuo, Li-li Guan, Ning Dai

**Affiliations:** Department of Gastroenterology, the first Affiliated Hospital of Dalian Medical University, 222, Zhongshan Road, Dalian, 116011 Liaoning Province China; Department of Digestive Physiology, Dalian Medical University, Dalian, Liaoning Province China

**Keywords:** Salidroside, Oxidative stress, Non-alcoholic steatohepatitis, CYP2E1, Nox2

## Abstract

**Background:**

Nonalcoholic steatohepatitis (NASH) is characterized by fat accumulation in the hepatocyte, inflammation, liver cell injury, and varying degrees of fibrosis, and can lead to oxidative stress in liver. Here, we investigated whether Salidroside, a natural phenolic antioxidant product, can protect rat from liver injury during NASH.

**Methods:**

NASH model was established by feeding the male SD rats with high-fat and high-cholesterol diet for 14 weeks. Four groups of male SD rats including, normal diet control group, NASH model group, and Salidroside treatment group with150mg/kg and 300 mg/kg respectively, were studied. Salidroside was given by oral administration to NASH in rats from 9 weeks to 14 weeks. At the end of 14 weeks, liver and serum were harvested, and the liver injury, oxidative stress and histological features were evaluated.

**Results:**

NASH rats exhibited significant increases in the following parameters as compared to normal diet control rats: fat droplets with foci of inflammatory cell infiltration in the liver. ALT, AST in serum and TG, TC in hepatocyte elevated. Oxidative responsive genes including CYP2E1 and Nox2 increased. Additionally, NASH model decreased antioxidant enzymes SOD, GSH, GPX, and CAT in the liver due to their rapid depletion after battling against oxidative stress. Compared to NASH model group, treatment rats with Salidroside effectively reduced lipid accumulation, inhibited liver injury in a does-dependent manner. Salidroside treatment restored antioxidant enzyme levels, inhibited expression of CYP2E1 and Nox2 mRNA in liver, which prevented the initial step of generating free radicals from NASH.

**Conclusion:**

The data presented here show that oral administration of Salidroside prevented liver injury in the NASH model, likely through exerting antioxidant actions to suppress oxidative stress and the free radical–generating CYP2E1 enzyme, Nox2 in liver.

## Background

Nonalcoholic steatohepatitis (NASH) is the progressive form of nonalcoholic fatty liver disease (NAFLD) and features of NASH on liver biopsy include steatosis, inflammation and varying degrees of fibrosis [1]. NASH is associated with obesity, type 2 diabetes and metabolic syndrome, and its increasing prevalence and clinical severity. Thus it is quickly becoming a significant public health concern [2].

While a variety of factors are involved in NASH development and pathogenesis, it is well-accepted that the development of NASH follows a two-hit model [3]. The “1^st^ Hit” involves excess lipid accumulation in the liver, which sensitizes the liver to the “2^nd^ Hit”. The “2^nd^ Hit” involves inflammation, oxidative stress, liver damage and fibrosis. This two-hit hypothesis is helpful in understanding processes that contribute to development and progression of NASH, the risk factors and underlying cellular and molecular mechanisms in NASH development remain largely undefined, which has limited the development of therapies to prevent/treat NAFLD/NASH.

Oxidative stress is thought to be a major contributor to the pathogenesis and progression of NASH [4]. Oxidative stress has been defined as an imbalance between oxidants and antioxidants in favor of the former, resulting in an overall increase in cellular levels of reactive oxygen species (ROS) [5]. In patients with histopathologically progressive NASH, production of antioxidants is reduced, and the total antioxidant capacity is apparently insufficient to compensate for oxidative stress [6]. Therefore, it is speculated that agents, such as vitamin E, that promote cellular antioxidant defense activity are likely to have therapeutic potentials in NASH prevention/treatment [7].

Salidroside (SDS, p-hydroxyphenethyl-b-D-glucoside) is a natural phenolic secondary metabolite from Rhodiola rosea L, which has been used as a herbal medicine for centuries. Salidroside, in particular, has potent antioxidation activities [8, 9]. Moreover, recent studies have shown that SDS has a great protective efficacy on liver disease via its antioxidant activities [10–12]. However, whether SDS can provide protection against NASH with oxidative stress remains unknown. Therefore, in this study, we used a liver oxidative stress model induced by high-fat and high-cholesterol (HFHC) diet in rats and evaluated the protective effects of SDS on NASH.

### Materials

#### Reagents

Salidroside [purity > 98] was provided by Sciphar Hi-tech Industry Co., Ltd, Shanxi, China, and were diluted in distilled water before use. High-fat and high-cholesterol (HFHC) diet (containing 15 % lard, 2 % cholesterol and 83 % normal diet) was provided by Department of Digestive Physiology at Dalian Medical University. Triglycerides (TG), Total cholesterol (TC) and malondialdehyde (MDA), glutathione (GSH) as well as superoxide dismutase (SOD), glutathione peroxidase (GPX), catalase (CAT) commercial testing kits were bought from JianCheng Bioengineering Institute, Nanjing, China.

## Methods

Male SD rats, weighing 140–160 g, 6 weeks of age, were provided by the Laboratory Animal Center at Dalian Medical University. Rats were housed individually. A normal laboratory diet and water were available *ad libitum*. The animal room was maintained at constant temperature of 23 ± 1 °C and 50 % relative humidity with a 12 h (7:00 a.m.–7:00 p.m.) light/dark cycle. Food was removed the night before the experiment. All experimental procedures were examined and approved by the Animal Care and Use Committee of Dalian Medical University (Dalian, China) and performed in strict accordance with the People’s Republic of China Legislation Regarding the Use and Care of Laboratory Animals. After 1 week on the normal diet, 40 animals were randomly divided into 4 groups:normal diet control rats (*n* = 10): rats were fed with normal diet for 14 weeks; NASH model group (*n* = 10): rats were fed with HFHC diet for 14 weeks; SDS-treated rats (*n* = 10, two groups): rats were fed with HFHC diet for 14 weeks with a daily oral feeding of SDS 150 mg/kg and 300 mg/kg respectively by intragastric (i.g.) gavage from 9 weeks to 14 weeks. After the end of 14 weeks, all rats were anesthetized at 24 h after the last treatment. Blood was collected by cervical decapitation and centrifuged at 1500 g for 20 min at 4 °C to obtain serum. Liver tissue was homogenized in ice-cold PBS buffer and centrifuged at 1800 g for 10 min at 4 °C to precipitate the insoluble material, and the supernatant was used in the following assays.

### Evaluation of liver pathology

Liver tissue sample from each rat were fixed in 10 % phosphate-buffered formalin, and then embedded in paraffin blocks. Five-micrometer tissue sections were cut and stained with hematoxylin and eosin (H&E) for histological analysis (NAS scoring) [13] under light microscope (Nikon, Japan). Briefly, the NAS score was aggregated from 4 semi-quantitative sub-scores: steatosis (0–3), lobular inflammation (0–2), hepatocellular ballooning (0–2), and fibrosis (0–4).

### Serum biochemical test

Alanine aminotransferase (ALT) and aspartate aminotransferase (AST) in serum were used as indicators of hepatocyte function and injury. ALT and AST levels were measured by a Bayer 1650 automatic analyzer (Germany).

### Triglyceride, total cholesterol detection in hepatocytes

For the determination of total cholesterol and triglycerides, liver samples (100 mg) were homogenized, and lipids were extracted with 2 ml of chloroform and methanol (2:1), as described by Folch et al [14]. Lipids were dissolved in 2 % Triton X-100 (Sigma, St. Louis, MO), as described by Carr et al [15]. Hepatic triglyceride and cholesterol levels were determined using commercially available reagents.

### Lipid peroxidation and antioxidative enzyme activity in liver

Malondialdehyde (MDA) and the enzymatic activities of superoxide dismutase (SOD), glutathione peroxidase (GPX), glutathione (GSH) as well as catalase (CAT) in liver were measured using commercial testing kits according to the manufacturer’s instructions. MDA and GSH levels are expressed as nmol/mg protein and mg/g protein, respectively. The enzymatic activity of the SOD, GPX, and CAT are expressed as U/mg protein.

### RNA isolation and quantitative real-time PCR analysis

CYP2E1 and NOX2 mRNA expression were tested by Quantitative Real-Time PCR. Total RNA was extracted from liver tissue samples by the TRIzol kit (Gibco/Life Technologies) according to the manufacturer’s protocol. The RNA was then reverse-transcribed to cDNA using the Super-Script II (Invitrogen) and the target genes were amplified using Power SYBR Green PCR Master Mix reagent (Applied Biosystems). The amplification was performed in real-time PCR system (Applied Biosystems) and a modified PCR cycles were used as following: initial denaturation at 95 °C for 2 min, followed by 35 cycles were performed at 95 °C for 30 s and 60 °C for 30 s. The housekeeping gene β-actin was used as an internal control, and gene-specific mRNA expression was normalized agains β-actin expression. Relative quantification by the 2^−ΔΔCT^ method was realized by comparing to control groups (The sequences for the primers used for Real time PCR: CYP2E1, GenBank ID: NM_031543, Forward-GACTGTGGCCGACCTGTT, Reverse- ACTACGACTGTGCCCTTGG; NOX2, GenBank ID: NM_001128123, Forward-TCAAGTGTCCCCAGGTATCC, Reverse-CTTCACTGG CTGTACCAAAGG; β-actin Forward-TGTCACCAACTGGGACGATA, Reverse- AACACA GCCTGG ATGGC AC).

### Statistical analyses

Differences among groups were examined by one-way ANOVA followed by Tukey-Kramer multiple comparison tests. Values are expressed as the mean ± SD. A value of *P* < 0.05 was considered statistically significant. All statistical analysis was performed by using SPSS software (version 11.0, SPSS, Inc.).

## Results

### Treatment with SDS prevents NASH-liver injury and steatosis

**Fig. 1:**
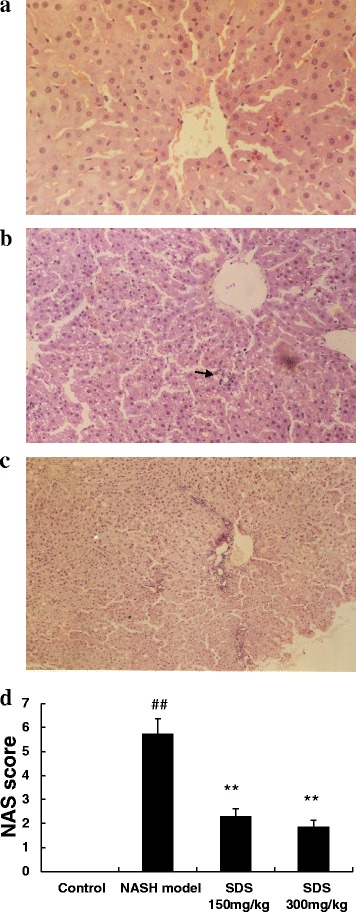
SDS improved histological features of NASH (1A-1C)- induced by high-fat and high-cholesterol diet in rats (**a**: normal H&E 100x magnification; **b**: NASH model H&E 100x magnification; **c**: SDS 300 mg/kg. H&E 100x magnification) and NAS score (1**d**). (Data are mean + SD, *n* = 10 per group. ##*P* < 0.01 compared to normal control; **P < 0.01 compared to NASH model)

**Fig. 2:**
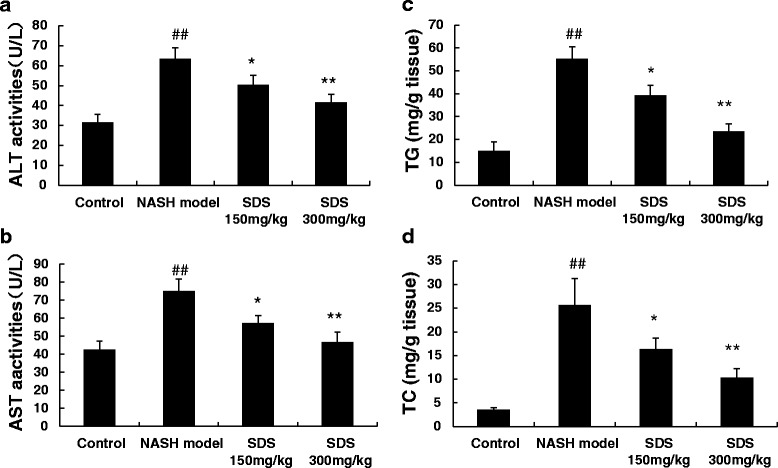
SDS (150 mg/kg and 300 mg/kg) dose-dependent decreased ALT (2**a**), AST (2**b**) in the serum and TG (2**c**), TC (2**d**) in the hepatocyte of NASH-induced by high-fat and high-cholesterol diet in rats. (Data are mean + SD, *n* = 10 per group. ##*P* < 0.01 compared to normal control; *P < 0.05, **P < 0.01 compared to NASH model)

To study the protective effects of SDS, we established a HFHC diet-induced NASH rats model. As shown in Fig. 1, liver steatosis that affected a large number of hepatocytes with foci of inflammatory cell infiltration throughout the lobule (arrow) was observed in H&E stained liver sections from HFHC diet rats. In contrast, the liver sections were normal from normal control rats (Fig. 1a, b). Also, significantly increased serum ALT, AST (Fig. 2a, b) and liver TG, TC were detected in HFHC diet rats (Fig. 2c, d). These results indicate that a three-weeks HFHC diet feeding sufficiently induced NASH in rats. Importantly, compared to NASH model group, a dramatic reduction both in lipid droplets and in the inflammatory infiltration were detected in the liver from SDS-treated group (Fig. 1c). Consistent to the observations from H&E staining, the average NASH scores were significantly reduced in HFHC diet rats (Fig. 1d). In addition, the levels of ALT, AST in the serum and TG, TC in the hepatocyte also decreased in the SDS-treated group when compared with those in the NASH model group, and SDS inhibited the levels of ALT, AST, TG and TC in a dose-dependent manner (Fig. 2a-d). These results demonstrate that SDS is an potential agent for prevention of rat from HFHC diet -induced NASH.

### SDS suppress liver injury–induced oxidative stress mediators

**Fig. 3:**
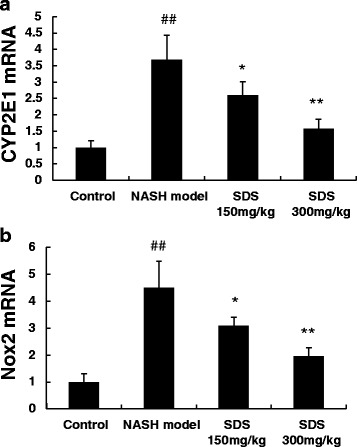
Dose-dependent inhibitory effects of SDS (150 mg/kg and 300 mg/kg) on hepatic CYP2E1 (**3a**) and Nox2 (**3b**) mRNA expression in liver with NASH-induced by high-fat and high-cholesterol diet in rats. (Data are mean + SD, *n* = 10 per group. ##*P* < 0.01 compared to normal control; *P < 0.05, **P < 0.01 compared to NASH model)

The imbalance between oxidative and anti-oxidative stress response has been well accepted as a critical pathogenic factor for NASH development. As SDS has an antioxidative activity, we speculated that SDS may protect rat from NASH through this antioxidative function. To test this hypothesis, we analyzed the expression of antioxidation enzymes. HFHC diet feeding resulted in a significant increase in MDA level, and largely decreased the antioxidation enzymes including SOD, GSH, GPX, and CAT in the liver as compared to the normal controls (Table 1). Similarly, the mRNA levels of CYP2E1 (Fig. 3a) and Nox2 (Fig. 3b) were also increased in HFHC diet rats liver. Interestingly, compared to untreated HFHC diet group, the SDS treatment significantly reduced MDA and increased SOD, GSH, GPX, and CAT levels in a dose dependent manner. Further analysis indicated that SDS dose-dependently suppressed CYP2E1 and Nox2 mRNA expression in the liver. Therefore, these results suggest that SDS can protect rat from NASH through inhibiting the liver oxidative stress.

**Table 1:** Effects of SDS on liver MDA and SOD, GSH, GPX, CAT contents in NASH rats

Group	MDA (nmol/mg prot)	SOD (U/mg prot)	GSH (mg/g prot)	GPX (U/mg prot)	CAT (U/mg prot)
Control	0.61 ± 0.22	101.51 ± 2.58	13.15 ± 2.65	456.76 ± 55.42	36.61 ± 6.71
NASH model	1.71 ± 0.23*	46.42 ± 9.31*	6.48 ± 1.25*	295.76 ± 64.32*	19.51 ± 8.06*
SDS treatment (150 mg/kg)	1.09 ± 0.20**	72.01 ± 3.38**	9.98 ± 1.62**	398.12 ± 39.13**	26.19 ± 5.73**
SDS treatment (300 mg/kg)	0.67 ± 0.18***	97.21 ± 3.52***	12.23 ± 1.32***	425.32 ± 49.23**	31.76 ± 5.32**

Data are mean + SD, *n* = 10 per group

*P < 0.01 *vs* Control group

**P < 0.05,***P < 0.01 *vs* NASH model group

## Discussion

Our preclinical studies demonstrated that SDS protects rat from HFHC diet -induced NASH through suppressing the oxidative stress-induced liver damages, as evidenced by the results that SDS significantly reduced the increased MDA and increased the suppressed antioxidation enzymes, like SOD, GSH, GPX, and CAT, in the NASH-injured liver. At the same time, SDS significantly reduced the increased CYP2E1and Nox2 mRNA expression in the liver. These results suggest that SDS can protect the liver from NASH-induced injury, which is most likely to take place through the inhibition of oxidative stress mediators. In this experiment, rats were treated by oral administration of salidroside from 9 weeks to 14 weeks. By the ninth week, mild to moderate pathology should be estabilished. Therefore, salidroside can recover the severe pathology after the full development of the pathology.

Oxidative stress is significantly closely associated with NASH. Antioxidants can ameliorate the development of NASH formation [16]. The current work revealed that SDS rendered the increased CYP2E1 less pronounced in injured liver with NASH in rats. The CYP2E1 enzyme is a hepatic cytochrome P_450_ isoform, which create free radicals in phase I enzymes [17]. CYP2E1 is critically important in NASH development by promoting oxidative, inflammation. CYP2E1 overexpression results in increased oxidative stress and nitrosative stress in mouse model of non-alcoholic fatty liver [18]. But CYP2E1-null mice can prevent NASH progression [19]. The fact that SDS can prevent the upregulation of the cytochrome P450 enzyme CYP2E1 suggests that SDS exert hepatoprotection by acting early in the process of oxidative stress, which is probably capable of blocking the entire cascade of the process that leads to liver injury and inflammation. However, the precise celluar and molecular mechanisms by which SDS bind to targets upstream of CYP2E1 remains to be elucidated.

The results of the current study also revealed that SDS treatment reduced the rate of upregulation of Nox2 mRNA expression in the NASH rat model. Nox2 is a membrane-bound enzyme complex. It has been shown to be involved in cellular respiratory bursts and free radical production in a variety of cells, including hepatocytes [20]. Nox-2-derived reactive oxygen species (ROS) may be involved in the activation of inflammatory apoptotic pathways. NOX2-generated oxidative stress is associated with severity of liver steatosis in patients with non-alcoholic fatty liver disease [21]. For this reason, Nox2 has been proposed as a potential therapeutic target to reduce ROS-related injury, such as ischemia-reperfusion associated liver injury. It has been reported that NO donor KMUP-1 improves hepatic ischemia-reperfusion and hypoxic cell injury by inhibiting Nox2- and reactive oxygen species (ROS)-mediated inflammation [22].

The CYP pathway is known to be coupled with the NOX pathway [23, 24]. The increased expression of CYP2E1 leads to the generation of more free electrons, which is coupled with the conversion of NADPH to NADP^+^ via Nox2 and/or Nox4. The CYP2E1 reaction cycle produces ROS as a result of uncoupling of the reaction. In addition, Nox2 and Nox4 may promote the recycling of NADP^+^ to produce superoxides and peroxides, which can further result in the generation of peroxides and ROS through the Fenton chemistry. These CYP2E1/NOX2-coupled reactions increase the caspase-3 activity, induce DNA fragmentation, and ultimately result in the apoptosis in liver tissues [20]. This is characterized by NASH-induced steatosis in liver. In this way, the Nox2 pathway is another therapeutic target for diseases that involve oxidative stress [25, 26]. The current results demonstrated that SDS can suppress the increased CYP2E1 and Nox2 expression, suggesting that SDS may exert the hepatoprotective effect through inhibition of the CYP2E1/Nox2 coupling reaction, reducing oxidative stress, and ameliorating liver injury caused by NASH. This is the first report to show that SDS prevent NASH via this mechanism of action.

## Conclusions

In summary, the current work provides evidence that SDS can prevent liver injury and steatosis in NASH rats by inhibiting oxidative stress. The action probably involves SDS exerting antioxidant actions and the inhibition of free radical–generating CYP2E1/Nox pathway. Although the research have no the highest dose normal control group, literature demonstrated that the dose (150 and 300 mg/kg SDS) used in this study did not pose genotoxic effect in vivo and in vitro [27].

## Abbreviations

ALT: alanine aminotransferase
AST: aspartate aminotransferase
CAT: catalase
CYP2E1: cytochromeP_450_2E1
GPX: glutathione peroxidase
GSH: glutathione
HFHC: high-fat and high-cholesterol
MDA: malondialdehyde
NAFLD: nonalcoholic fatty liver disease
NASH: nonalcoholic steatohepatitis
Nox2: nicotinamide adenine dinucleotide phosphate oxidase2
ROS: reactive oxygen species
SDS: salidroside
SOD: superoxide dismutase
TC: total cholesterol
TG: triglycerides

## References

[1] Ludwig J, Viggiano TR, McGill DB, Oh BJ (1980). Nonalcoholic steatohepatitis: Mayo Clinic experiences with a hitherto unnamed disease. Mayo Clin Proc.

[2] Charlton MR, Burns JM, Pedersen RA, Watt KD, Heimbach JK, Dierkhising RA (2011). Frequency and outcomes of liver transplantation for nonalcoholic steatohepatitis in the United States. Gastroenterology.

[3] Day CP, James OF (1998). Steatohepatitis: a tale of two “hits”?. Gastroenterology.

[4] Koek GH, Liedorp PR, Bast A (2011). The role of oxidative stress in non-alcoholic steatohepatitis. Clin Chim Acta.

[5] Sies H (1997). Oxidative stress: oxidants and antioxidants. Exp Physiol.

[6] Sreekumar R, Rosado B, Rasmussen D, Charlton M (2003). Hepatic gene expression in histologically progressive nonalcoholic steatohepatitis. Hepatology.

[7] Sanyal AJ, Chalasani N, Kowdley KV, McCullough A, Diehl AM, Bass NM, Neuschwander-Tetri BA, Lavine JE, Tonascia J, Unalp A, Van Natta M, Clark J, Brunt EM, Kleiner DE, Hoofnagle JH, Robuck PR, NASH CRN (2010). Pioglitazone, vitamin E, or placebo for nonalcoholic steatohepatitis. N Engl J Med.

[8] Yu P, Hu C, Meehan EJ, Chen L (2007). X-ray crystal structure and antioxidant activity of salidroside, a phenylethanoid glycoside. Chem Biodivers.

[9] Dhar P, Bajpai PK, Tayade AB, Chaurasia OP, Srivastava RB, Singh SB (2013). Chemical composition and antioxidant capacities of phytococktail extracts from trans-Himalayan cold desert. BMC Complement Altern Med.

[10] Wu YL, Lian LH, Jiang YZ, Nan JX (2009). Hepatoprotective effects of salidroside on fulminant hepatic failure induced by D-galactosamine and lipopolysaccharide in mice. J Pharm Pharmacol.

[11] Wu YL, Piao DM, Han XH, Nan JX (2008). Protective effects of salidroside against acetaminophen-induced toxicity in mice. Biol Pharm Bull.

[12] Yuan Y, Wu SJ, Liu X, Zhang LL (2013). Antioxidant effect of salidroside and its protective effect against furan-induced hepatocyte damage in mice. Food Funct.

[13] Brunt EM, Kleiner DE, Wilson LA, Belt P, Neuschwander-Tetri BA, NASH Clinical Research Network (CRN) (2011). Nonalcoholic fatty liver disease (NAFLD) activity score and the histopathologic diagnosis in NAFLD: distinctclinicopathologic meanings. Hepatology.

[14] Folch J, Lees M, Sloane Stanley GH (1957). A simple method for the isolation and purification of total lipides from animal tissues. J Biol Chem.

[15] Carr TP, Andresen CJ, Rudel LL (1993). Enzymatic determination of triglyc-eride, free cholesterol, and total cholesterol in tissue lipid extracts. Clin Biochem.

[16] Korish AA, Arafah MM (2013). Camel milk ameliorates steatohepatitis, insulin resistance and lipid peroxidation in experimental non-alcoholic fatty liver disease. BMC Complement Altern Med.

[17] Lee GH, Bhandary B, Lee EM, Park JK, Jeong KS, Kim IK, Kim HR, Chae HJ (2011). The roles of ER stress and P450 2E1 in CCl4-induced steatosis. Int J Biochem Cell Biol.

[18] Kathirvel E, Chen P, Morgan K, French SW, Morgan TR (2010). Oxidative stress and regulation of anti-oxidant enzymes in cytochrome P4502E1 transgenic mouse model of non-alcoholic fatty liver. J Gastroenterol Hepatol.

[19] Abdelmegeed MA, Banerjee A, Yoo SH, Jang SH, Gonzalez FJ, Song BJ (2012). Critical role of cytochrome P450 2E1 (CYP2E1) in the development of high fat-induced nonalcoholic steatohepatitis. J Hepatol.

[20] Shah A, Kumar S, Simon SD, Singh DP, Kumar A (2013). HIV gp120- and methamphetamine-mediated oxidative stress induces astrocyte apoptosis via cytochrome P450 2E1. Cell Death Dis.

[21] Del Ben M, Polimeni L, Carnevale R, Bartimoccia S, Nocella C, Baratta F, Loffredo L, Pignatelli P, Violi F, Angelico F (2014). Nox2-generated oxidative stress is associated with severity of ultrasound liver steatosis in patients with non-alcoholic fatty liver disease. BMC Gastroenterol.

[22] Kuo KK, Wu BN, Chiu EY, Tseng CJ, Yeh JL, Liu CP, Chai CY, Chen IJ (2013). NO donor KMUP-1 improves hepatic ischemia-reperfusion and hypoxic cell injury by inhibiting oxidative stress and pro-inflammatory signaling. Int J Immunopathol Pharmacol.

[23] Eid AA, Gorin Y, Fagg BM, Maalouf R, Barnes JL, Block K, Abboud HE (2009). Mechanisms of podocyte injury in diabetes: role of cytochrome *P*450 and NADPH oxidases. Diabetes.

[24] Wang X, Ke Z, Chen G, Xu M, Bower KA, Frank JA, Zhang Z, Shi X, Luo J (2012). Cdc42-dependent activation of NADPH oxidase is involved in ethanol-induced neuronal oxidative stress. PLoS One.

[25] Cairns B, Kim JY, Tang XN, Yenari MA (2012). Nox inhibitors as a therapeutic strategy for stroke and neurodegenerative disease. Curr Drug Targets.

[26] Sorce S, Krause KH, Jaquet V (2012). Targeting Nox enzymes in the central nervous system: therapeutic opportunities. Cell Mol Life Sci.

[27] Zhu J, Wan X, Zhu Y (2010). Evaluation of salidroside in vitro and in vivo genotoxicity. Drug Chem Toxicol.

